# Molecular Imaging and Theragnostics of Thyroid Cancers

**DOI:** 10.3390/cancers14051272

**Published:** 2022-03-01

**Authors:** Luca Giovanella, Desiree’ Deandreis, Alexis Vrachimis, Alfredo Campenni, Petra Petranovic Ovcaricek

**Affiliations:** 1 Clinic for Nuclear Medicine and Molecular Imaging, Imaging Institute of Southern Switzerland, Ente Ospedaliero Cantonale, 6500 Bellinzona, Switzerland; 2 Division of Nuclear Medicine, Department of Medical Sciences, AOU Città della Salute e della Scienza, University of Turin, 10126 Turin, Italy; desiree.deandreis@unito.it; 3 Department of Nuclear Medicine, German Oncology Center, University Hospital of the European University, Limassol 4108, Cyprus; alexis.vrachimis@goc.com.cy; 4 Nuclear Medicine Unit, Department of Biomedical and Dental Sciences and Morpho-Functional Imaging, University of Messina, 98121 Messina, Italy; acampenni@unime.it; 5 Department of Oncology and Nuclear Medicine, University Hospital Center Sestre Milosrdnice, 10000 Zagreb, Croatia; p.petranovic@gmail.com

**Keywords:** thyroid cancer, molecular imaging, theragnostics, radioiodine, positron emission tomography

## Abstract

**Simple Summary:**

According to the American Cancer Society, approximately 53,000 new cases of thyroid cancer were diagnosed and more than 2200 people died from the disease in 2020. New developments in molecular imaging are significantly improving thyroid cancer diagnostics and therapy. Continuous research in molecular imaging techniques additionally contributes to an understanding of a variety of diseases and enables more efficient care of thyroid cancer patients. Molecular imaging-based personalized therapy has been a fascinating concept for individualized therapeutic strategy, which is able to attain the highest efficacy and reduce adverse effects in certain patients. Theragnostics, which integrates diagnostic testing to detect molecular targets for particular therapeutic modalities, is one of the key technologies that contributes to the success of personalized medicine. This review details the inception of molecular imaging and theragnostic applications for thyroid cancer management.

**Abstract:**

Molecular imaging plays an important role in the evaluation and management of different thyroid cancer histotypes. The existing risk stratification models can be refined, by incorporation of tumor-specific molecular markers that have theranostic power, to optimize patient-specific (individualized) treatment decisions. Molecular imaging with varying radioisotopes of iodine (i.e., ^131^I, ^123^I, ^124^I) is an indispensable component of dynamic and theragnostic risk stratification of differentiated carcinoma (DTC) while [^18^F]F-fluorodeoxyglucose ([^18^F]FDG) positron emission tomography/computed tomography (PET/CT) helps in addressing disease aggressiveness, detects distant metastases, and risk-stratifies patients with radioiodine-refractory DTC, poorly differentiated and anaplastic thyroid cancers. For medullary thyroid cancer (MTC), a neuroendocrine tumor derived from thyroid C-cells, [^18^F]F-dihydroxyphenylalanine (6-[^18^F]FDOPA) PET/CT and/or [^18^F]FDG PET/CT can be used dependent on serum markers levels and kinetics. In addition to radioiodine therapy for DTC, some theragnostic approaches are promising for metastatic MTC as well. Moreover, new redifferentiation strategies are now available to restore uptake in radioiodine-refractory DTC while new theragnostic approaches showed promising preliminary results for advanced and aggressive forms of follicular-cell derived thyroid cancers (i.e., peptide receptor radiotherapy). In order to help clinicians put the role of molecular imaging into perspective, the appropriate role and emerging opportunities for molecular imaging and theragnostics in thyroid cancer are discussed in our present review.

## 1. Introduction

Thyroid cancers account for about 1% of all cancer cases, being the most frequent endocrine malignancy. Among cancers derived from the follicular thyroid cell, the most frequent (80–85% of cases) differentiated thyroid cancer (DTC) usually has a favorable prognosis while the anaplastic form (ATC) evolves rapidly to the fatal outcome in approximately all cases [1]. The 5-year disease-specific mortality rate of poorly differentiated thyroid carcinoma (PDTC) and medullary thyroid carcinoma (MTC), a neuroendocrine tumor derived by the parafollicular C-cells, is ~50% [2]. Surgery, when technically feasible, represents the mainstay of treatment of most thyroid cancer [3]. Postoperative radioiodine therapy (RAI) may be administered in DTC patients to ablate thyroid remnants, reduce the risk of recurrence and disease-related death or treat known structural disease [4]. Postoperative monitoring of most DTC and MTC patients relies on serum tumor markers and neck ultrasound, with additional imaging added in cases of suspicious spread of the disease [5,6]. Treatment of relapsing and advanced DTC may benefit from RAI while Thyrosin-Kinase Inhibitors (TKI) can be employed in patients affected by advanced radioiodine-refractory DTC and MTC, respectively [1,7,8]. This review focuses on how molecular imaging influences the diagnosis, staging, prognostic stratification, and management of thyroid cancers. Additionally, the role of molecular imaging-based theragnostics applications is also discussed.

## 2. Diagnosis of Thyroid Cancers

Thyroid nodules are very common in the adult population, while the cancer rate among people with a thyroid nodule is rare. Accordingly, screening for thyroid cancer is not recommended [9]. Ultrasound (US) is a first-line tool for the initial malignancy risk assessment [10,11], and suspicious nodules are addressed with Fine Needle Aspiration (FNA). The main limitations of thyroid FNA, however, are “indeterminate” nodules. The rate of malignancy ranges from 10 to 30% in such cases, with histological examination a necessity to achieve the final diagnosis [12]. Molecular imaging methods can fruitfully contribute to refining the preoperative diagnosis of indeterminate thyroid nodules [13,14].

### 2.1. Molecular Imaging of Thyroid Nodules

Thyroid scintigraphy (TS), performed using either [^99m^Tc]Pertechnetate (Na[^99m^Tc]TcO_4_) or [^123^I]Sodium-Iodide (Na[^123^I]I) is the only method able to detect autonomously functioning thyroid nodules (AFTNs) and exclude malignancy with a 96–99% negative predictive value (NPV) even in the presence of low-normal TSH levels [15]. Moreover, molecular imaging with [^99m^Tc]Tc-hexakis-(2-methoxy-2-isobutyl isonitrile ([^99m^Tc]Tc-MIBI) and [^18^F]FDG may help to differentiate benign from malignant indeterminate nodules [14,15].

#### 2.1.1. [^99m^Tc]Tc-MIBI Thyroid Scintigraphy

The [^99m^Tc]Tc-MIBI is a lipophilic cation able to cross the cell membrane. It penetrates into the cytoplasm in a reversible way and then irreversibly moves through the membrane of the mitochondria using a different electrical gradient regulated by a high negative inner membrane electric potential. Tumor cells are characterized by a higher negative inner membrane electric potential compared to normal cells. Consequently, it can be helpful to characterize the biological behavior of cytologically indeterminate nodules [16]. A very high to absolute NPV was found in thyroid nodules with a [^99m^Tc]Tc-MIBI hypoactive pattern, while an increased [^99m^Tc]Tc-MIBI uptake conferred a significantly higher risk of cancer. However, while a NPV of >96 is achieved, the positive predictive value (PPV) is unsatisfactory as [^99m^Tc]Tc-MIBI uptake may also be recorded in benign nodules (especially follicular and oxyphilic adenomas) [17,18]. Moreover, a semiquantitative approach to evaluate the tracer washout from the nodule (Washout Index (WOind)) was introduced, providing better results in comparison with qualitative analysis [19].

#### 2.1.2. [^18^F]FDG Positron Emission Tomography/Computed Tomography

[^18^F]FDG uptake is related to an overexpression of the transmembrane glucose transporter proteins (GLUTs), which transport the tracer into the cell, and to the overactivation of hexokinases that phosphorylate [^18^F]FDG to [^18^F]FDG-6-phosphate and trap the tracer in the cell. Interestingly, a visually [^18^F]FDG-negative indeterminate thyroid nodule has a negligible risk of malignancy, making [^18^F]FDG PET/CT a suitable ruling-out test (as robustly demonstrated by meta-analyses [20,21,22]). Moreover, [^18^F]FDG PET/CT radiomics analysis preliminarily proved to increase specificity and PPV [23].

Table 1 gives an overview of the different tracers and imaging methods to further evaluate thyroid nodules in a clinical setup.

## 3. Differentiated Thyroid Cancers

The current strategy for DTC management is a risk-stratified approach based on information from surgical histopathology and molecular markers, post-operative serum thyroglobulin (Tg) levels, and functional/anatomical imaging studies.

### 3.1. Surgical Treatment for DTC and Preoperative Staging

Traditionally, (near-) total thyroidectomy was performed in most DTC patients, even though the current American Thyroid Association (ATA) guidelines recommend lobectomy for patients with unifocal intrathyroidal low-risk DTC [5]. Cervical lymph nodal metastases occur in 20–60% of patients with DTC, and central and/or lateral neck compartment dissection reduces the risk of local-regional recurrence. Prophylactic central neck dissection may improve regional control for invasive tumors (T3–T4), but it is discouraged for low-risk DTC because of potential associated morbidities are not justified by a significant clinical benefit [5]. Preoperative neck US generally suffices to plan surgery, however additional cross-sectional imaging (i.e., contrast-enhanced computed tomography (ceCT), magnetic resonance imaging (MRI)) are reserved for patients with locally advanced disease or for those that are at a high risk of developing distant metastases [24]. PET/CT with [^18^F]FDG could be performed preoperatively in aggressive DTC and anaplastic thyroid cancer (see the specific section). Kim et al. retrospectively analyzed 60 patients with low- or intermediate-risk DTC who underwent [^18^F]FDG PET/CT before thyroidectomy. They reported very low sensitivity and NPV in detecting lymph-node metastases (10% and 50%, respectively) but very high specificity (90%) [25]. Some other studies compared the accuracy of PET with that of neck ultrasound and ceCT. The specificities of PET, ultrasonography, and ceCT in evaluating both the central and lateral neck regions were very high. However, the sensitivities were low (≤50%) for all three modalities. The overall diagnostic accuracy of [^18^F]FDG PET/CT tended to be higher for lateral than for central lymph nodes [26,27].

### 3.2. Postoperative ^131^I Therapy

Initial (near-)total thyroidectomy followed by ^131^I administration has remained the mainstay in achieving a cure in many DTC patients. ^131^I treatment of DTC is based on the principle of sodium iodide symporter (NIS) expressing thyroid cells with DTC cells having the ability of trapping circulating ^131^I. After surgery, the risk of structural disease recurrence and/or persistence is assessed using the three-tier (low, intermediate, high) stratification recommended by ATA [5]. The goal of therapeutic ^131^I administration after total thyroidectomy is outlined based on standardized definitions as follows [5,24].

- Remnant ablation to eliminate normal thyroid tissue remnants in low risk patients, thereby ensuring undetectable or minimal serum Tg levels (in the absence of neoplastic tissue), which facilitates follow-up.
- Adjuvant treatment to irradiate suspected but unproven sites of neoplastic cells in low-intermediate and intermediate risk patients, as determined by histopathologic features, thereby reducing the risk of disease recurrence.
- Treatment of known disease to treat persistent or recurrent disease in patients with demonstrated metastatic disease.

Basically, two approaches to ^131^I therapy planning and delivery are adopted in clinical practice: (i) the approach based on clinical-pathologic factors and institutional protocols (i.e., risk-adapted approach), and (ii) the approach integrating postoperative radioiodine functional imaging (i.e., functional imaging-guided). No evidence data are available to allow recommending one strategy over the other and the local choice is based on local factors (i.e., surgery quality, laboratory and imaging availability, and multidisciplinary thyroid team), including physician–patient preferences.

### 3.3. The Role of Functional Imaging in Managing Radioiodine Therapy

Nuclear imaging of DTC takes advantage of the NIS-mediated uptake allowing characterization of iodine-avid versus noniodine-avid disease, as well as the localization and quantification of iodine-avid postoperative thyroid remnant.

#### 3.3.1. Post-Therapy Whole-Body Scintigraphy (TxWBS)

When DTC patients receive ^131^I therapy based on clinical-pathological risk stratification a [^131^I] post-treatment whole-body scintigraphy (TxWBS) is obtained 3–10 days after treatment. TxWBS is a highly sensitive and specific diagnostic tool useful to determine the location and extent of iodine-avid thyroid tissue. Consequently, it permits accurate disease restaging by detecting unknown loco-regional and/or distant metastases thus changing the initial risk stratification and customizing additional therapies and follow-up strategies. The diagnostic performance of TxWBS can be significantly improved using additional single-photon emission computed tomography/computed tomography (SPECT/CT). Compared to planar imaging, SPECT/CT images (i) reveal more foci of pathological radioiodine uptake located either in the cervical region or at distance (i.e., higher sensitivity), (ii) distinguish physiological uptakes from the foci of the disease (i.e., higher specificity), and (iii) detect metastatic lesions in unexpected sites [28,29].

**Figure 1:**
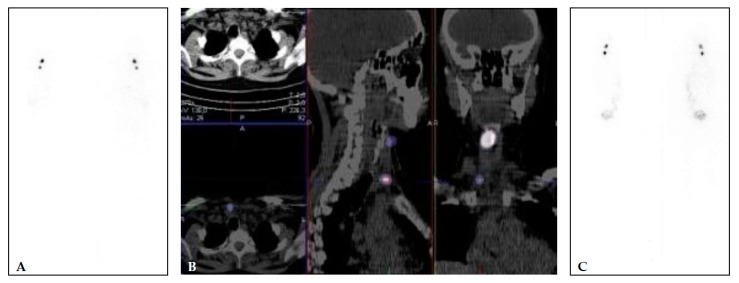
(**A**,**B**) 40 years old female, with multifocal papillary microcarcinoma (left lobe 7 mm and 2 foci of 1 mm in the right lobe (pT1a(m)N0). TSH 67.83 mIU/L, Tg 11.83 μg/L (<2 μg/L), and TgA 2.2 kIU/L (<4.5 kIU/L). Diagnostic WBS and SPECT/CT (**A**,**B**) performed after application of 74 MBq of [^131^I] show two focuses of [^131^I] uptake—larger in the right upper part of the region VI and small focus lower in the region VI, right paratracheal. The patient was treated with 1665 MBq (45 mCi) of [^131^I]. Post-treatment [^131^I] WBS (**C**) shows an accumulation of the [^131^I] in the same two focuses.

Figure 1 illustrates a patient that received both pre-ablational WBS with a diagnostic dose and its correlative after therapeutic dose (TxWBS).

#### 3.3.2. Postoperative Diagnostic Whole-Body Scintigraphy (DxWBS)

**Figure 2:**
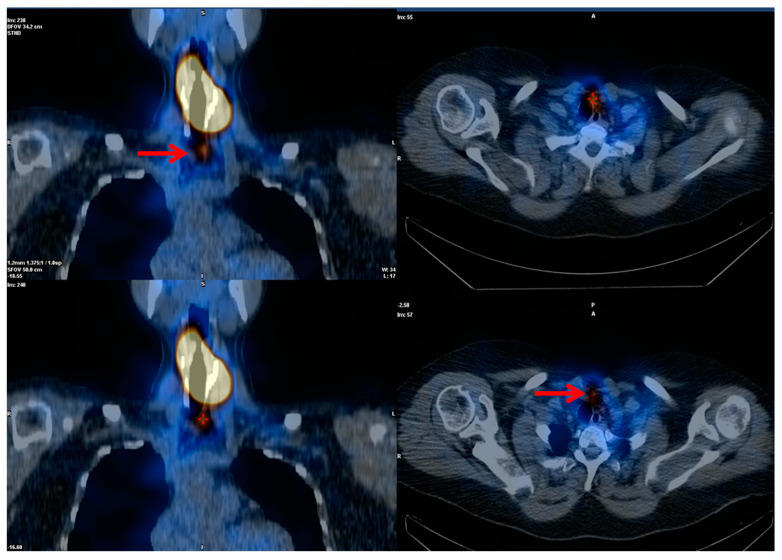
Post-treatment high-activity ^131^I whole-body scintigraphy. Large thyroid remnant with a small single iodine-avid lymph node metastasis (red arrows) at central neck compartment.

**Figure 3:**
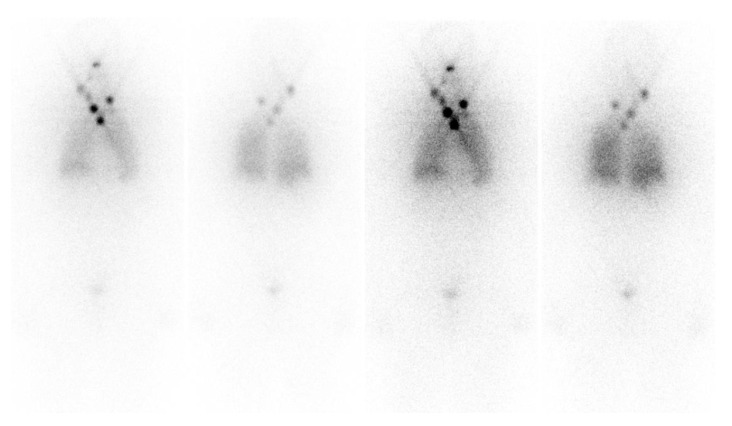
Post-treatment high-activity ^131^I whole-body scintigraphy. Multiple cervical and mediastinal iodine-avid metastatic lymph nodes and diffuse lung “miliariform” metastases.

When the functional-imaging guided approach is preferred by the local team, postoperative whole-body scintigraphy with diagnostic activities of different radioactive iodine isotopes ([^131^I], [^123^I], [^124^I]) is performed. Theoretically, postoperative DxWBS leads to a significant improvement of risk stratification and staging of DTC patients and informs subsequent [^131^I] therapy [30]. As an example, visualization of metastatic lesions prompts risk re-stratification and, potentially, adjustment of [^131^I] administered activity. Indeed, the suspicion of noniodine-avid disease (i.e., negative radioiodine WBS with elevated thyroglobulin (Tg) or Tg levels out of proportion to the WBS findings) may require additional studies (i.e., neck/chest computed tomography, CT; [^18^F]FDG PET/CT). Finally, negative WBS results with undetectable Tg levels may rule out ^131^I therapy in low-risk DTC patients. Some authors, however, argue that TxWBS is more sensitive in identifying metastatic lesions (not initially seen on DxWBS), avoiding the so-called “stunning effect” of iodine-avid tissue [31]. Notably, recent improvements in technology (i.e., SPECT/CT), image acquisition, and reconstruction protocols enabled the use of lower [^131^I] activities [32]. Accordingly, a systematic review of 14 original research articles describing the incremental value of [^131^I] SPECT/CT demonstrated significant clinical benefit in terms of staging, risk stratification, and follow-up of DTC [33]. Figure 2 and Figure 3 show examples of [^131^I] TxWBS in patients with lymphogenous and/or distant spread.

In addition, [^123^I] and/or [^124^I] can be used instead of ^131^I to minimize the risk of stunning. Pre-ablation [^123^I] WBS provided additional critical information in 25% of 122 patients, by revealing unsuspected regional or distant metastases and thus guiding the administration of higher ^131^I therapeutic activities, or revealing unexpected large thyroid remnants [34].

Other authors also demonstrated that the information gained by [^123^I] DxWBS changed the applied [^131^I] activity in 49% of cases [35]. Santhanam and colleagues, in a recent meta-analysis, described a 94% sensitivity of [^124^I] PET/CT in postoperative detection of metastatic lesions amenable to RAI [36]. In particular, it allows lesion-based evaluation of iodine uptake and clearance that is especially advisable to tailor [^131^I] therapy in patients with advanced disease [37,38]. Finally, new perspectives in the field are represented by NIS-imaging via [^18^F]tetrafluoroborate ([^18^F]TFB) and [^18^F]fluorosulfate ([^18^F]FSO_3_). The visualization of the expression of NIS through in vivo molecular imaging has been based for a number of years on diagnostic or post-therapeutic [^131^I] WBS. The limitations of scintigraphic methods, including resolution and the possibility of target quantification, are well known. The technique of choice in the diagnostic setting could be [^124^I] PET/CT, as it shows higher sensitivity compared to the [^131^I] WBS, but also allows for dosimetric estimation in therapeutic cases. However, it is not easily available and for its intrinsic characteristic requires a long time to scan with (presently) suboptimal quality and resolution of images. Recently, new tracers such as [^18^F]TFB or [^18^F]FSO_3_ have shown promise as potential candidates for NIS visualization in preclinical studies and first preliminary human applications [39]. From a technical point of view, the development of fluorinate tracers has the advantages of easier labeling, higher image quality, and high tumor to background contrast in animal and human studies. From a biological point of view [^18^F]TFB and [^18^F]FSO_3_ are iodine analogues, but with a main difference, the formers do not go into the iodine organification process in thyroid cells. These radiopharmaceuticals have been tested in healthy volunteers with promising biodistribution, in particular, low background uptake in the liver, muscle, and brain and high uptake in organs that normally express NIS (thyroid, salivary gland, and stomach) [40]. Furthermore, a rapid blood clearance (20 min after injection) and stability up to 4 h after injection have been observed. In breast cancer mice models [^18^F]TFB compared to [^123^I] whole-body scan showed superior imaging characteristics related to a faster blood clearance, higher tumor/blood ratio, and higher sensitivity related to PET imaging [41]. O’Doherty et al. evaluated [^18^F]TFB uptake in 5 patients with intrathyroidal thyroid cancer while Samnik et al. compared [^18^F]TFB to [^124^I] PET in 9 patients after total thyroidectomy [42,43]. In the first study, intra-thyroid tumor nodules showed lower uptake compared to normal thyroid tissue, while in the second the two tracers showed almost comparable performance. In only 2 patients, [^18^F]TFB showed two more lesions compared to [^124^I] PET and in all cases lower retention in thyroid tissue, probably due to the absence of the organification phase. Their applications in thyroid cancer patients still require further exploration and at this moment are under investigation in pilot studies (NCT03196518). Of note, any therapeutic [^131^I] administration should be followed by a TxWBS to assess therapeutic [^131^I] localization, which is routinely used together with a preablation Tg measurement, to complete post-operative staging and predict the patient’s outcome.

#### 3.3.3. [^18^F]FDG Positron Emission Tomography/Computed Tomography (PET/CT)

Very promising results of [^18^F]FDG PET/CT performed at the time of first postoperative staging were reported in patients with high-risk DTC and Hürthle cell and poorly differentiated histotypes. In particular, [^18^F]FDG PET/CT might be very useful in correctly addressing disease aggressiveness and in detecting distant metastases (especially bone) in such cases [44].

### 3.4. The Role of Functional Imaging in Response Assessment and Disease Monitoring

Thyroglobulin measurement and ultrasound are essential tools to monitor DTC after primary treatment [5,24,45,46].

#### 3.4.1. Radiodine Whole Body Scintigraphy

There is no consensus regarding the routine use of DxWBS during the follow-up of patients with DTC [47]. Indeed, DxWBS remains a useful tool in selected patients with (i) higher risk of persistent/recurrent disease, (ii) extra-thyroid radioiodine uptake at TxWBS (i.e., loco-regional and/or distant metastases), (iii) poorly informative TxWBS (i.e., large remnants), or (iv) positive thyroglobulin antibody values. Gonzalez Carvalho and colleagues [48] evaluated a very large cohort of DTC patients (*n* = 1420) in all TNM categories in whom a life-long follow-up (up to 25 years) was regularly performed also using DxWBS. They concluded that in DTC patient follow-up, the use of DxWBS can still be justified at least until stimulated thyroglobulin is below functional sensitivity (in the absence of thyroglobulin antibodies) and DxWBS is negative (i.e., no evidence of radioiodine loco-regional and/or distant uptake). Importantly, the results of a large SEER database (28,220 patients diagnosed with DTC between 1998 and 2011) showed that follow-up DxWBS performed after primary treatment of DTC are the only imaging studies associated with improved disease-specific survival [49].

#### 3.4.2. Positron Emission Tomography

**Figure 4:**
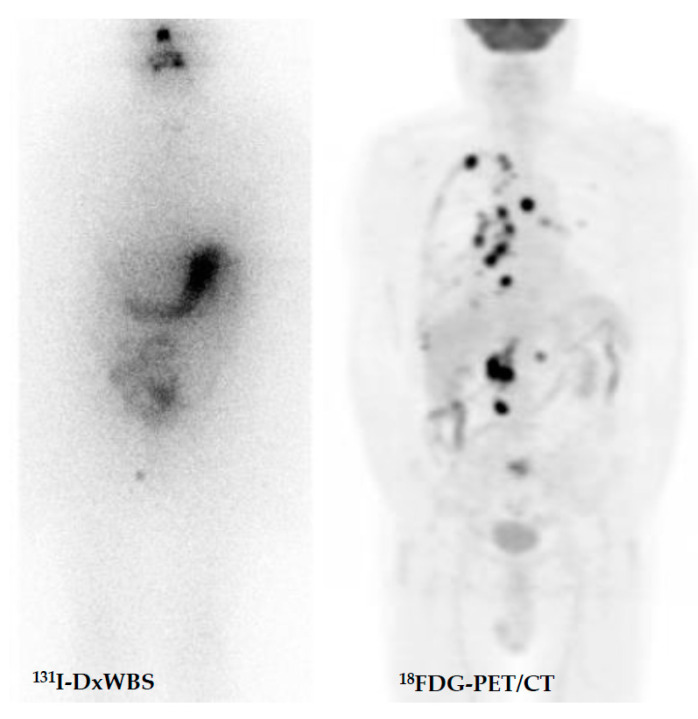
DTC patient with increasing thyroglobulin and negative diagnostic whole-body scan after thyroidectomy and radioiodine therapy. [^18^F]FDG-avid relapsing disease.

The main indication for [^18^F]FDG PET/CT in DTC is during DTC follow-up in the case of high or increasing thyroglobulin levels, but negative or doubtful ultrasound, and negative diagnostic and post-therapeutic RAI imaging. In such a context, evidence supports the use of [^18^F]FDG PET/CT, with reported pooled sensitivity and specificity of 80% and 90%, respectively [50,51,52]. Figure 4 demonstrates a patient with this flip flop phenomenon (i.e., WBS negative and FDG positive). The current ATA guidelines suggest that [^18^F]FDG PET/CT should be performed when stimulated thyroglobulin levels are >10 ng/mL. Further, although the positivity rate of [^18^F]FDG PET/CT increases with higher serum thyroglobulin level, true-positive results have also been reported in 10–20% of DTC patients with serum thyroglobulin levels <10 ng/mL [53,54]. Giovanella et al. suggested that shortened thyroglobulin doubling time (i.e., <1 year) independently predicted a positive [^18^F]FDG PET/CT scan in patients with biochemical recurrence [55]. Considering the relatively low sensitivity of diagnostic ^131^I WBS, one of the most important issues is the comparison of post-therapeutic ^131^I WBS with [^18^F]FDG PET/CT. Leboulleux et al. evaluated the sensitivity of post-therapeutic [^131^I] WBS versus [^18^F]FDG PET/CT in patients with elevated serum thyroglobulin levels. The sensitivity in detecting DTC relapse was 88% for [^18^F]FDG PET/CT and 16% for TxWBS (*p* < 0.01), respectively. [^18^F]FDG-PET/CT was abnormal in 22 patients, five of whom also had an abnormal TxWBS. The authors concluded that in patients with suspicion of recurrence based on thyroglobulin levels after a normal TxWBS, [^18^F]FDG PET/CT is better able to localize disease than TxWBS [56]. Similarly, Kim et al. reported that second empiric ^131^I therapy and TxWBS were neither diagnostically nor therapeutically useful in 39 patients with elevated stimulated thyroglobulin and negative [^18^F]FDG PET/CT after initial treatment. These data suggest that the correct integration of radioiodine imaging and [^18^F]FDG PET/CT may optimize additional administrations of high [^131^I] activities and inform alternative strategies (i.e., surgical procedures or external beam radiotherapy) [57].

## 4. Advanced and Radioactive Iodine Refractory Cancer

Radioactive iodine is the main treatment modality in the case of metastatic disease that accounts for around 10% of patients with thyroid cancer. About 50% of metastatic patients obtain complete remission or stabilization on a long-term period after RAI therapy. Unfortunately, in the remaining patients, disease does not achieve complete or partial response or progress despite RAI treatment [58]. According to ATA guidelines, RAI-refractory (RAI-R) thyroid cancer is defined in the following situations: (1) the structurally known disease does not ever concentrate RAI (no uptake outside the thyroid bed at the first TxWBS); (2) the tumor tissue loses the ability to concentrate RAI after previous evidence of RAI-avid disease; (3) RAI is concentrated in some lesions but not in others; (4) metastatic disease progresses despite significant uptake of RAI [5]. Such definitions, however, cannot be merely applied to all cases—each patient should be individually managed with a good understanding of the limitations of the various classifications [59]. Further classifications related to scarce response to RAI include: [^18^F]FDG avid lesions at PET/CT, disease progression from 12 to 16 months after RAI, and incomplete response after >22.2 GBq of cumulative activity of RAI [60,61,62]. There is increasing evidence that treatment response in advanced disease is strictly related to the absorbed doses delivered to tumors and healthy organs, leading to the need for personalized therapy. Dosimetric evaluation could be provided by both maximum tolerated activity (MTA) calculation or lesional dosimetry. The first approach has the main goal to administer higher activities of radioactive iodine limiting toxicity, while respecting the absorbed dose limit to the blood marrow of 2 Gy and whole-body retention of 80 mCi at 48 h [63]. Nevertheless, higher activities could not be effective if a significant absorbed dose is not delivered to the tumors. To overcome this issue, lesional dosimetry has the main goal to predict, when performed before therapy, or to assess, when performed after treatment, the absorbed dose in each single lesion to modulate administered activities [64]. Lesional dosimetry is normally performed by diagnostic/post-therapeutic [^131^I] SPECT/CT or [^124^I] PET/CT imaging and demonstrates a high inter-lesion and inter-patient heterogeneity, allowing easy identification of lesions that are less likely to respond to RAI. Furthermore, it is not feasible in all lesions and presents some limits in the case of disseminated metastases. Accordingly, further studies are needed to estimate the impact of dosimetry on patients’ progression-free survival or overall survival [65,66,67,68]. Beyond RAI uptake as it is, several factors (i.e., biological/molecular basis of thyroid cancer, specific patterns of patients, and disease presentation) should be taken into account and considered more deeply on a case by case basis [69]. In a clinical setting, [^18^F]FDG PET/CT is a useful tool as a prognostic and predictive factor of RAI treatment response in metastatic patients. Deandreis et al., in a retrospective study including eighty patients with metastatic thyroid cancer showed that [^18^F]FDG PET/CT uptake was the only significant prognostic factor for survival (*p* = 0.02), and the prognosis was significantly related both to SUVmax and to the number of [^18^F]FDG avid lesions (*p* = 0.03 and 0.009, respectively). Furthermore, patients with both [^18^F]FDG and RAI avid lesions have a prognosis similar to [^18^F]FDG only avid lesions [70]. More recently Manohar et al. demonstrated in 62 RAI refractory DTC metastatic patients that [^18^F]FDG PET/CT should be considered as an imaging biomarker predictive for overall (OS) and progression free survival (PFS), and that tumor burden represented by higher than median values of metabolic tumor volume and total lesion glycolysis were associated with worse OS (*p* = 0.06) and PFS (I = 0.007) [71].

### Medical Treatments and Re-Differentiation Strategies

The therapeutic management of RAI-R thyroid cancer depends on several considerations, in particular the patient status, the extension and site of the disease, and the assessment of disease progression. Beyond RAI, TKI represent the main systemic treatments, in progressive and high tumor burden disease, to improve patients’ outcomes. Timing to define disease as “progressive” is around 12–16 months according to the inclusion criteria in clinical trials and usually based on RECIST 1.1 criteria. Sorafenib and Lanvatinib, a multitarget TKI, have demonstrated a major improvement in progression-free survival in phase III clinical trials [72,73]. Several other drugs are under investigation in clinical trials, with some of the most promising results coming from apatinib, an inhibitor of VEGFR [74], and from TKI treatments in thyroid cancer with rarer fusion mutations such as larotectinib in TRK mutate tumors in basket trials [75]. Nevertheless, with these latest drug types, there is increasing evidence of the possibility to re-differentiate RAI-R cancers through molecular blockage induced by targeted drug direct effect [76]. The first-in-human trial, from Ho et al. on the basis of pre-clinical study in BRAF mutated mouse models, showed the potential of selumetinib to reinduce a significant [^131^I] uptake through the inhibition of MAPK cascade [77]. Radioactive iodine uptake was restored in 12/20 included patients. The 8/12 that reached a presumed absorbed dose of 2000 cGy were treated and achieved objective response in all cases (5 partial responses (PR) and 3 stable diseases (SD)). Nevertheless, RAI uptake reinduction was observed in only 4/9 patients harboring BRAF mutation and only 1 patient among them was treated with [^131^I] and achieved an objective response. Consequently, specific BRAF inhibitors (i.e., dabrafenib, vemurafenib, or trametinib) that most likely induce a sustained MAPK pathway inhibition are tested as single agents or in combination, in re-differentiation clinical trials in BRAF mutated tumors [78]. Finally, one of the treatment options which has been recently proposed for RAI-R thyroid cancer has been peptide receptor radionuclide therapy (PRRT), based on the theranostic concept. PRRT is a unique way of targeting somatostatin receptors overexpression on tumor cells in many cancers including the thyroid [79]. In an early study in which [^68^Ga]Ga-DOTA-TOC PET/CT was used to select such patients for PRRT as well as to assess treatment response and toxicity, PR and SD were seen in approximately 60% of the treated patients, with duration of the response being 3.5–11.5 months [80].

Recently, Maghsoomi et al., reviewed available studies investigating the safety and efficacy of PRRT in patients with advanced radioiodine refractory DTC and metastatic medullary thyroid carcinoma (see the specific section). Out of 2284 related papers, 41 were included in the analysis according to the inclusion criteria. A total of 157 RAI-R DTC patients were treated with PPRT. Biochemical and objective responses (partial and complete) were observed in 25.3 and 10.5% of patients, respectively [81]. The main adverse events were nausea, asthenia, and transient hematologic toxicity. Currently, however, such results cannot be treated as sufficient evidence for recommending this form of treatment in RAI-R DTC. However, PRRT should be selectively considered in patients with advanced radioiodine refractory DTC with progression under established treatment (i.e., TKI, see below).

## 5. Poorly Differentiated Thyroid Cancer

Poorly differentiated thyroid cancer (PDTC) is rare, with a reported incidence from 2% to 15% of all thyroid cancers [82,83]. PDTC is generally considered a thyroid cancer entity with a prognosis between DTC and anaplastic thyroid cancer (ATC) due to its higher risk of persistence/recurrence both locally and distantly, and higher mortality [84]. As compared to DTC, PDTC generally occurs in older ages and has a higher prevalence in men. Most patients have an extrathyroidal extension at presentation and 50–85% of patients are detected with lymph nodal spread in the regional lymph nodes, whereas the vast majority (85%) develop distant spread in the course of the disease [85]. Like DTC, total thyroidectomy and central and/or lateral neck dissection in the presence of suspicious lymph nodes is the cornerstone of multimodal treatment. The five-year overall survival for PDTC is in the range of 62–85% and the disease-specific survival is 66%. Poor outcome predictors are age (>45 years), locally advanced disease (including extrathyroidal extension), mitosis, and necrosis [86].

### Molecular Imaging and Theragnostics

**Figure 5:**
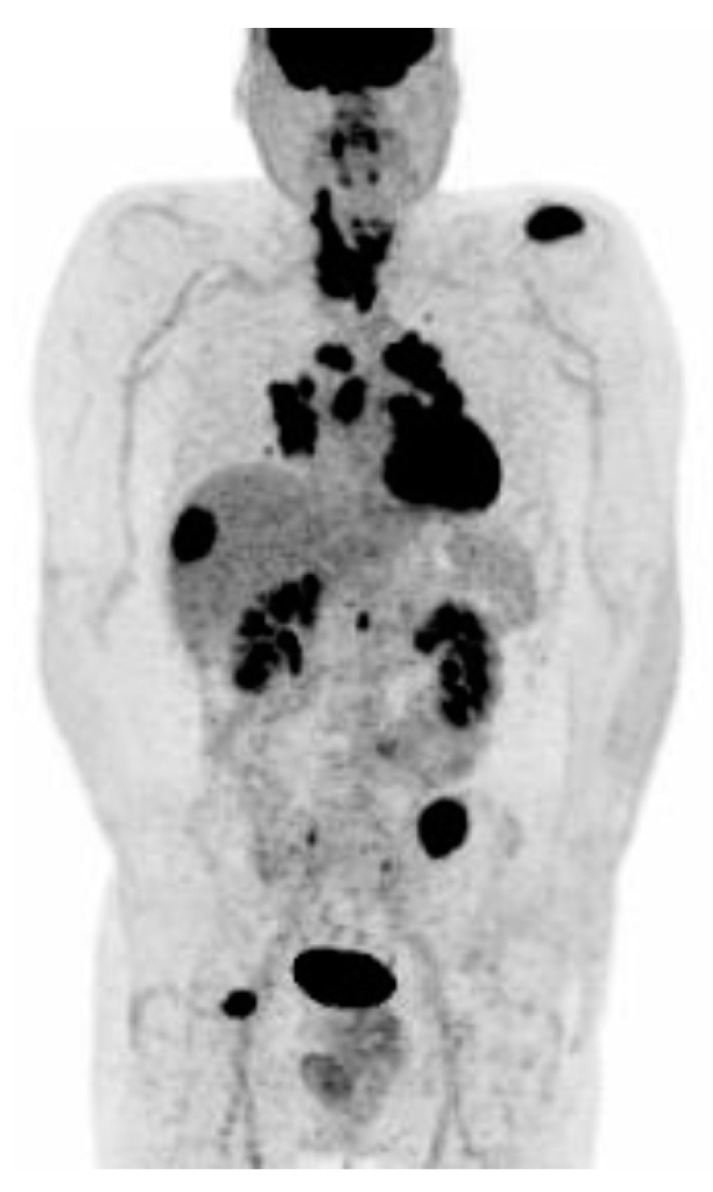
Advanced radioiodine-refractory and [^18^F]FDG-avid metastatic thyroid carcinoma involving the thyroid “in toto” with multiple metastases (lymph nodes, lung, liver, kidney, and bone).

Correct staging with both [^18^F]FDG PET/CT and RAI imaging is mandatory to assess PDTC. Basically, postoperative RAI treatment should be performed because in many cases at least part of the disease shows a significant RAI uptake, which will reduce the tumor burden. On the other hand, high metabolic tumor volume (MTV) and total lesion glycolysis (TLG) in [^18^F]FDG PET/CT are significantly associated with poor prognosis [87]. Figure 5 demonstrates an example of PDTC measured with [^18^F]FDG. In such cases, PDTC is often, or will become, iodine-refractory. The current regimen of treatment in such cases is shifting toward targeting multiple pathways simultaneously to avoid (early) resistance to TKIs and/or produce a synergistic treatment effect.

## 6. Anaplastic Thyroid Carcinoma

**Figure 6:**
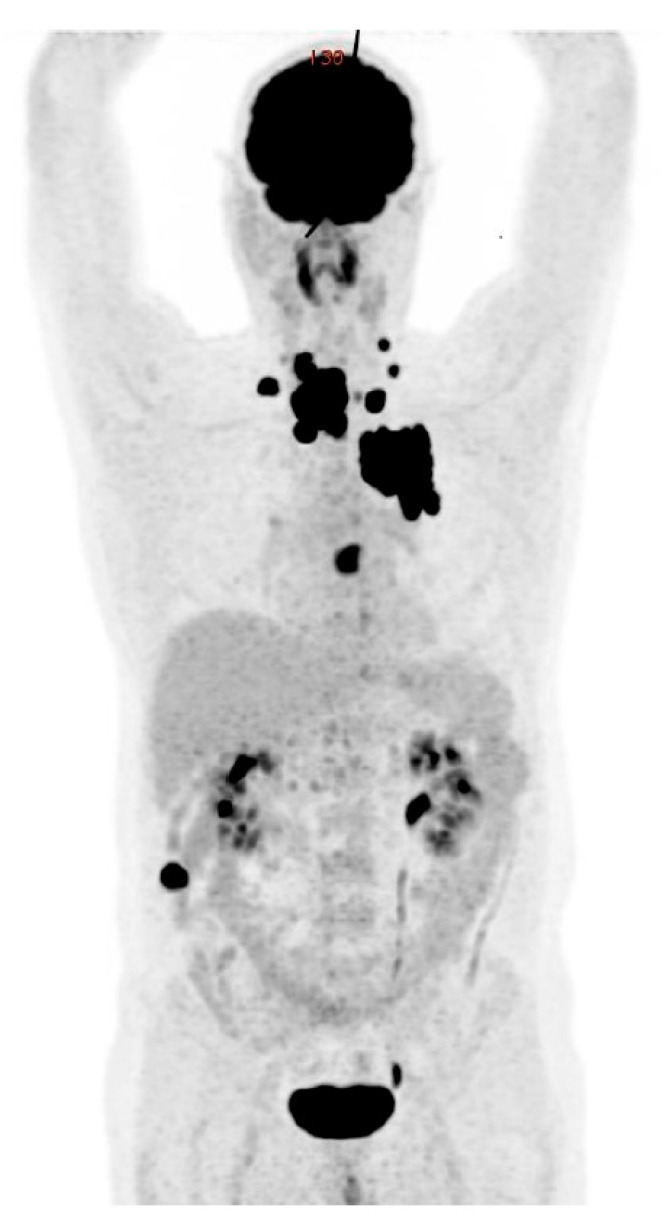
[^18^F]FDG-PET/CT: anaplastic thyroid cancer with diffuse loco-regional invasion, lymph node, and distant metastases (lung, heart, and bone).

Anaplastic thyroid carcinoma (ATC) is the most aggressive, yet most rare, thyroid cancer type. It accounts for 1% of all thyroid cancers, with an estimated incidence of 1–2 cases per year per million population. This undifferentiated form of thyroid cancer is unable to take up RAI and is in most cases lethal [88,89]. So far, no established treatment is available that prolongs survival despite published guidelines for the management of ATC [90]. ATC presents a very low median overall survival (5–6 months), and the median one-year survival is not more than 20%. It is associated with a rapid and lethal progression, especially at a local level with the noncontrollable locoregional disease, with an invasion of vital structures in the neck being the major cause of death [91]. [^18^F]FDG PET/CT could be useful in identifying the true spread of the disease, which could further guide treatment (Figure 6). It is still unclear whether patients will benefit from debulking surgery, due to the high rate of complications [92]. With regards to radiation, a large analysis of nearly 1300 non-resected ATC patients demonstrated a significantly improved survival in patients that had undergone radiotherapy. However, if this could be translated to other patients after surgery as well, is still unclear. In small cohorts, the addition of chemotherapy to radiotherapy has led to an improvement in overall survival. Whether chemotherapy or combination radiochemotherapy improves patients’ outcome is still unclear [93]. Recently, dabrafenib (BRAF inhibitor) and trametinib (MEK inhibitor) have been approved for inoperable locally invasive or metastatic ATC.

### Molecular Imaging and Theragnostics

High SUVmax, MTV, and TLG in [^18^F]FDG PET/CT are significantly associated with poor prognosis [94]. Accordingly, correct staging and risk stratification with [^18^F]FDG PET/CT have a major clinical impact on the management as they will identify patients that will profit from a more aggressive multimodal treatment, ideally as part of a clinical trial [95,96,97]. Furthermore, new molecular targets such as prostate-specific membrane antigen (PSMA) have shown increased uptake in anaplastic thyroid cancers. Thus, radioligand therapies with beta or alpha emitters could prove beneficial in selected cases that experience a high expression of molecular targets [98].

## 7. Medullary Thyroid Cancer

Medullary thyroid carcinoma (MTC) is a neuroendocrine tumor that accounts for less than 5% of malignant thyroid tumors. It is derived from parafollicular C cells of the thyroid. Approximately 75% of cases are considered sporadic, while the rest are hereditary [99]. Sporadic MTCs are mostly unifocal tumors, while hereditary tumors are multifocal, usually affecting both thyroid lobes [100] and occur as familial MTC (FMTC) or a part of multiple endocrine neoplasia syndromes type 2 (MEN2A and MEN2B). MEN2A accounts for 95% of MEN2 syndromes with four identified variants: classical MEN2A (MTC sometimes associated with pheochromocytoma, or hyperparathyroidism, or both), MEN2A with cutaneous lichen amyloidosis, MEN2A with Hirschsprung’s disease, and familial MTC (MTC without pheochromocytoma or hyperparathyroidism) [6]. Hereditary cases are induced by different germline mutations in the rearranged during transfection (RET) protooncogene (chromosomal band 10q11.2), while sporadic cases are caused by somatic RET mutations in up to 65% of cases [101,102,103].

### 7.1. Diagnosis

MTC is still recognized in the advanced stage without a significant shift toward early disease detection. MTC stage III and IV are present in almost 50% of patients at initial diagnosis. Ten-year survival rates of patients with stage I, II, III, or IV (AJCC/UICC TNM classification) are 100%, 93%, 71%, and 21%, respectively [6]. MTC is a morphologically very heterogeneous tumor. Neck ultrasound is useful in the evaluation of malignancy-risk of thyroid nodules. Hypoechoic texture with coarse calcifications is suspicious for MTC, however, it is not specific enough [2]. FNA cytology is obligatory in all cases with suspicious thyroid nodules. Calcitonin measurement in FNA washout of thyroid nodules and neck nodes has higher sensitivity compared to cytology in diagnosing MTC, and thus, it may eliminate false-negative results by cytology. Calcitonin measurement in needle washout is recommended for all patients with high serum calcitonin undergoing thyroid nodule biopsy [104].

The main serum tumor biomarker used in the diagnosis and follow-up of MTC is calcitonin, the secretory product of C-cells. It is an important diagnostic, as well as a prognostic, biomarker. Its serum levels are related to the content of C-cells. Furthermore, the measurement of serum calcitonin is also valuable in metastatic MTC to evaluate the response to systemic therapy [105]. MTC also secretes carcinoembryonic antigen (CEA) in half of all cases. However, it is not sensitive enough for diagnostic purposes or early assessment following initial surgery [106]. Procalcitonin, the precursor of calcitonin, shows even superior results compared to calcitonin in the management of MTC [107,108,109].

All MTC patients should undergo a neck ultrasound to evaluate local invasion and lymph node metastases, while additional morphological and functional imaging methods are recommended in patients with locally advanced and metastatic disease, as well as in all patients with a calcitonin level above 500 pg/mL [6,110,111]. Some studies reported high sensitivity of [^18^F]FDOPA PET/contrast-enhanced CT (PET/ceCT) in the initial staging of MTC patients before surgery. In addition, the sensitivity of [^18^F]FDOPA PET/ceCT demonstrated higher sensitivity in the detection of cervical lesions compared to neck US [112]. Increased biodistribution of the tracer in the liver decreases its sensitivity for liver lesions. In such cases, liver MRI may be advantageous [113]. Tracers targeting somatostatin receptors (e.g., [^68^Ga]Ga-DOTA-TATE) have shown promising results in the detection of MTC. Their accuracy is higher in MTC patients with increasing tumor markers. Important advantages are availability and the ability to provide a theragnostic approach in patients with lesions showing high expression of somatostatin receptors [114,115].

### 7.2. Surgical Treatment and Postoperative Management

Total thyroidectomy and risk-adapted cervical lymph node dissection is the main potentially curative option for patients with MTC, either sporadic or hereditary form [116]. Postoperative risk stratification of MTC patients is based on histopathological features, i.e., tumor stage, the loco-regional, and distant metastases [6,110,117]. Calcitonin is a useful prognostic tumor marker in postoperative follow-up. CEA is also useful as a biomarker of MTC recurrence and disease progression. The biochemical remission is possible in more than 50% of patients with an initial calcitonin concentration of ≤1000 pg/mL, but not in patients with calcitonin levels above 10,000 pg/mL [118]. Not only absolute calcitonin and CEA concentration but also their doubling time is an even stronger prognostic factor. Shorter doubling time negatively influences recurrence-free and overall survival [119]. Furthermore, serum Progastrin-releasing peptide (ProGRP) accurately discriminates MTC patients with loco-regional and distant metastasis. In addition, ProGRP outperforms calcitonin and CEA in monitoring the response of MTC to vandetanib therapy [120].

### 7.3. Molecular Imaging and Theragnostics

In post-operative management, imaging procedures are guided by tumor markers. Morphological imaging modalities are often falsely negative or inconclusive in patients with suspected persistent or recurrent MTC. Therefore, various functional imaging methods, targeting different metabolic pathways or receptors may be useful, including PET with [^18^F]FDG, [^18^F]FDOPA, and somatostatin analogs labeled with [^68^Ga]. Increased [^18^F]FDG uptake in MTC cells positively correlates with high expression of GLUTs and higher activity of hexokinase. Additionally, [^18^F]FDG uptake positively correlates with high proliferation and poor differentiation of tumor cells. Therefore, this imaging method may be more useful in patients with the aggressive disease [121]. The diagnostic performance [^18^F]FDOPA PET or PET/CT in patients with suspected recurrent MTC was evaluated in a meta-analysis of 8 studies including 146 patients. The diagnostic rates (DRs) of [^18^F]FDOPA PET/(CT) in per patient-based and lesion-based analyses were 66% and 71%, respectively. With the exclusion of PET-alone studies per patient-based DR increased to 72%. Detection rates also increased with high serum calcitonin concentrations of ≥1000 ng/L (86%) and short calcitonin doubling times of <24 months (86%) [122]. A recent study evaluated the diagnostic performance of different imaging modalities in 36 MTC patients with increased serum calcitonin levels and without known distant metastases [123]. Patient-based DRs of [^18^F]FDOPA PET/CT, [^18^F]FDG PET/CT, whole-body MRI, and whole-body CT were 64%, 40%, 40%, and 48%, respectively, demonstrating superior sensitivity of [^18^F]FDOPA PET/CT in localization of the recurrent and persistent disease. Asa et al. in a recent comparative study that enrolled 46 MTC patients with elevated calcitonin and/or CEA serum levels, evaluated the performance of [^18^F]FDOPA PET/CT and [^68^Ga]Ga-DOTA-TATE in the detection of recurrent or metastatic MTC [124]. [^18^F]FDOPA PET/CT demonstrated sensitivity, specificity, positive predictive value (PPV), NPV, and accuracy of 86.8%, 100%, 100%, 61.5%, and 89.1%, respectively, while [^68^Ga]Ga-DOTA-TATE PET/CT had a similar performance characteristics of 84.2%, 87.5%, 96.9%, 53.8%, and 84.6%, respectively.

**Figure 7:**
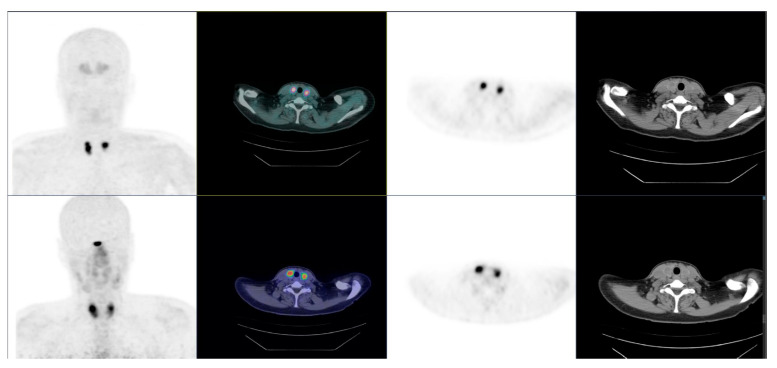
Bifocal and bilateral medullary thyroid carcinoma. Pre-operative PET/CT: primary tumors positive at [^18^F]FDOPA (**top**) and [^18^F]F-SiFAlinTATE (**bottom**).

**Figure 8:**
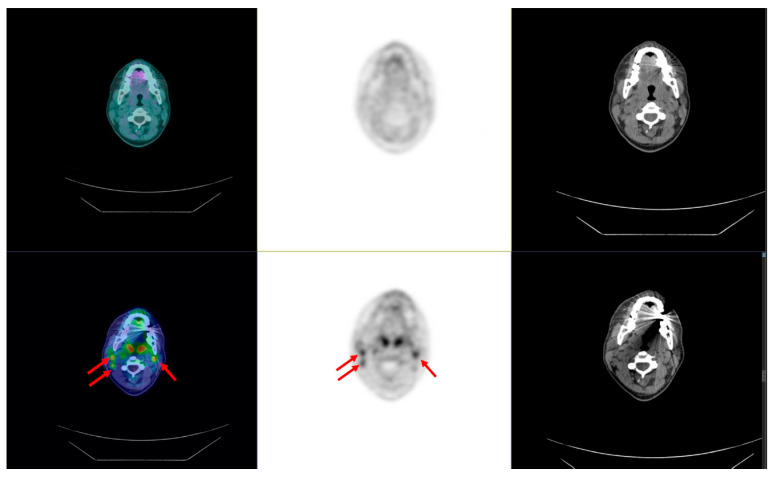
Bifocal and bilateral medullary thyroid carcinoma. Preoperative PET/CT: arrows showing bilateral lymph node metastases negative at [^18^F]FDOPA (**top**) and positive at [^18^F]F-SiFAlinTATE (**bottom**).

[^68^Ga]Ga-DOTA-TATE was superior in the detection of skeletal disease, while [^18^F]FDOPA PET/CT had better performance in the detection of liver and regional lymph node metastases. Thus, those two imaging modalities appear to be complementary. Figure 7 and Figure 8 show examples of different PET imaging of MTC.

Surgery, imaging-guided local treatments (i.e., external beam radiotherapy, thermal ablations, and cementoplasty), and systemic therapy (e.g., TKI, immune checkpoint inhibitors (ICPI), PRRT) can be used and combined to treat progressive advanced MTC [111]. Two multikinase inhibitors (cabozantinib and vandetanib) are approved for the therapy of progressive MTC as they result in stable disease or partial response, improving the progression-free survival of MTC patients. Cabozantinib showed higher clinical efficacy for patients with *RET* M918T or *RAS* mutation [125]. However, neither cabozantinib nor vandetanib prolonged the OS of patients with advanced MTC [126,127]. Additionally, both drugs may cause significant grade III or IV adverse events [128]. Immune checkpoint inhibitors targeting PD-L1/PD-1 may be useful in advanced disease, according to several studies that showed PD-L1 expression positively correlates with advanced MTC [129,130]. Radionuclide therapy with radiolabelled somatostatin analogs (SSAs) is another effective theragnostic option in selected cases. The expression of somatostatin receptors has been demonstrated in vitro [131] and in vivo studies [132]. This represents the basis for PRRT, mainly performed with [^177^Lu] and [^90^Y] radiolabelled SSAs. Grossrubatscher et al. showed in their review article that PPRT with SSTAs may accomplish disease control rate in 62.4% of cases; comprising of complete response in 2.6%, partial response in 5.1%, and stable disease in 54.7%. Notably, the majority of selected patients were in progression before starting with PPRT. Discontinuation of treatment due to toxicity is reported in only 1.3% of cases, hemotoxicity grade III–IV in 6.5% of cases, and nephrotoxicity grade III–IV in 0.8% [128]. Maghsoomi et al. in a systematic review among 220 patients with metastatic MTC demonstrated biochemical and objective responses (partial and complete) in 37.2 and 10.6% of patients, respectively [81]. Satapathy et al. showed that the disease control rate could be even higher (86%) if combining PRRT with a radiosensitizer, e.g., capecitabine [133]. Current studies also demonstrated progression-free or overall survival benefits [134,135]. Notably, the overall survival was not improved with other systemic therapies. Currently, PRRT with SSAs provides an alternative therapeutic option for MTC patients with SSTR positive metastatic disease that does not respond to other conventional therapies. MTC also shows the expression of cholecystokinin 2 receptors, which is a promising new target for PPRT therapy. These receptors are highly expressed in more than 90% of MTC cases [136]. Although very promising in theory, the clinical translation of cholecystokinin 2 receptor-targeted PRRT is hindered by a short in vivo half-life of the used radiopeptides. Simultaneous application of endopeptidase inhibitors is suggested to reduce their degradation in vivo, as endopeptidases are the main enzymes involved in the catabolism of cholecystokinin-based peptides [137].

## 8. Conclusions

Ultrasound, nuclear medicine, and molecular imaging play a relevant role in the management of thyroid cancer. In patients with thyroid nodules, an ultrasound assessment should be performed and used to select nodules for FNA. The integration of thyroid scintigraphy in the management plan is useful to exclude AFTN, which should not undergo FNA irrespective of the ultrasound indication. The contribution of thyroid scintigraphy with [^99m^Tc]Tc-MIBI and PET/CT with [^18^F]FDG is also relevant in patients with cytologically indeterminate nodules.

Risk stratification is pivotal in surgical, nuclear, and molecular treatment planning for DTC. The existing risk stratification models can be refined, by incorporation of tumor-specific molecular markers that have theranostic power, to optimize patient-specific (individualized) treatment decisions. RAI imaging is an indispensable component of dynamic and theranostic risk stratification, while [^18^F]FDG PET/CT helps in addressing disease aggressiveness, detects distant metastases, and risk-stratifies patients with RAI-R DTC, PDTC, and anaplastic cancers. Furthermore, [^124^I] PET/CT may guide a lesion-based dosimetric approach in patients submitted to RAI, with promising results. For MTC, [^18^F]FDOPA PET/CT and/or [^18^F]FDG PET/CT can be used depending on serum marker levels and kinetics. Finally, in addition to RAI, some theragnostic approaches are promising for metastatic MTC (SSAs- and cholecystokinin 2 receptor-targeted PRRT). New redifferentiation strategies are now available to restore uptake in RAI-R cancers, while new theragnostics approaches showed promising preliminary results for advanced and aggressive forms of follicular-cell derived thyroid cancers (SSAs-targeted PRRT, PSMA-internal radiotherapy).

## Figures and Tables

**Table 1:** Molecular imaging to differentiate benign and malignant thyroid nodules.

Method	Tracer	Indication	Pattern	Action
Scintigraphy	Na[^99m^Tc]TcO_4_	nodules-low TSH	AFTNs	Avoid FNA
Scintigraphy	Na[^123^I]I	nodules-low TSH	AFTNs	Avoid FNA
Scintigraphy	[^99m^Tc]Tc-MIBI	nodules-CI	[^99m^Tc]Tc-MIBI-	Avoid surgery
PET/CT	[^18^F]FDG	nodules-CI	[^18^F]FDG-	Avoid surgery

Legend: PET/CT, positron emission tomography/computed tomography; [^99m^Tc]Tc-MIBI, methoxy-isobutyl-isonitrile; [^18^F]FDG, fluorodeoxyglucose; TSH, thyrotropin; CI, cytologically indeterminate; AFTNs, autonomously functioning thyroid nodules; FNA, fine-needle aspiration.
